# Two RNAs or DNAs May Artificially Fuse Together at a Short Homologous Sequence (SHS) during Reverse Transcription or Polymerase Chain Reactions, and Thus Reporting an SHS-Containing Chimeric RNA Requires Extra Caution

**DOI:** 10.1371/journal.pone.0154855

**Published:** 2016-05-05

**Authors:** Bingkun Xie, Wei Yang, Yongchang Ouyang, Lichan Chen, Hesheng Jiang, Yuying Liao, D. Joshua Liao

**Affiliations:** 1 Guangxi Institute of Animal Sciences, Guangxi Key Laboratory of Livestock Genetic Improvement, Nanning, Guangxi, 530001, P.R. China; 2 Guangxi Veterinary Research Institute, Nanning, Guangxi, P.R. China; 3 Hormel Institute, University of Minnesota, Austin, Minnesota, 55912, United States of America; 4 College of Animal Science and Technology, Guangxi University, Nanning, 530004, P.R. China; 5 Department of Pathology, Guizhou Medical University Hospital, Guizhou, Guiyang, 550004, P.R. China; The John Curtin School of Medical Research, AUSTRALIA

## Abstract

Tens of thousands of chimeric RNAs have been reported. Most of them contain a short homologous sequence (SHS) at the joining site of the two partner genes but are not associated with a fusion gene. We hypothesize that many of these chimeras may be technical artifacts derived from SHS-caused mis-priming in reverse transcription (RT) or polymerase chain reactions (PCR). We cloned six chimeric complementary DNAs (cDNAs) formed by human mitochondrial (mt) 16S rRNA sequences at an SHS, which were similar to several expression sequence tags (ESTs).These chimeras, which could not be detected with cDNA protection assay, were likely formed because some regions of the 16S rRNA are reversely complementary to another region to form an SHS, which allows the downstream sequence to loop back and anneal at the SHS to prime the synthesis of its complementary strand, yielding a palindromic sequence that can form a hairpin-like structure.We identified a 16S rRNA that ended at the 4^th^ nucleotide(nt) of the mt-tRNA-leu was dominant and thus should be the wild type. We also cloned a mouse Bcl2-Nek9 chimeric cDNA that contained a 5-nt unmatchable sequence between the two partners, contained two copies of the reverse primer in the same direction but did not contain the forward primer, making it unclear how this Bcl2-Nek9 was formed and amplified. Moreover, a cDNA was amplified because one primer has 4 nts matched to the template, suggesting that there may be many more artificial cDNAs than we have realized, because the nuclear and mt genomes have many more 4-nt than 5-nt or longer homologues. Altogether, the chimeric cDNAs we cloned are good examples suggesting that many cDNAs may be artifacts due to SHS-caused mis-priming and thus greater caution should be taken when new sequence is obtained from a technique involving DNA polymerization.

## Introduction

**Fig 1:**
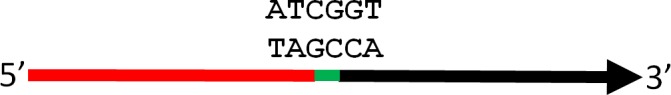
Illustration of SHS: Sequences of two different genes (coded by red and black bars, respectively) join together. At, and only at, the junction site (green bar), the two sequences are reversely complementary to each other, say 5’ATCGGT3’ *vs* 3’TAGCCA5’, while the rest parts of the two genes are not related to each other. In most cases this homologous region is short in length, and thus is coined as short homologous sequence, or SHS.

In various DNA and RNA sequence databases, there are tens of thousands of expression sequence tags (ESTs) of human origin that are chimeric complementary DNAs (cDNAs), dubbed chimeras herein[1,2]. For example, our bioinformatic analysis in 2013 identified 34770 chimeric ESTs in the National Center for Biotechnology Information (NCBI) of the United States [3], which is slightly more than the 31,005 chimeric ESTs identifed by Li et al in 2009 [4]. Each of these chimeras is formed by fusion of two different genes that are coined herein as “two partners” of the chimera. Li et al found that in 51.5% of these human chimeric ESTs, the two partners are fused at a short homologous sequence (SHS) of four or more nucleotides (nts) [4], and we found that 23252 of 34770 (67%) human chimeric ESTs contain an SHS, regardless of its length[3]. In an SHS-containing chimera, the 5’ partner has its 3’ region reversely complementary to the 5’ sequence of the 3’ partner, as illustrated in Fig 1, althought the two partner genes are not related. By our estimation, the length of an SHS is about six nts on average, although it varies greatly among different chimeras [3]. Moreover, the recent swift spread of RNA sequencing technology has also led to identification oftens of thousands of putative chimeric RNAs, especially in cancers[1]. After analyzing transcripts from about 1% of the human genome, the ENCODE project estimates that RNA transcripts from about 65% of the human genes form chimeras[5,6]. These lines of information seem to imply that chimeric RNAs are omnipresent in human cells, at least in cancer cells. However, the majority of the chimeras identified by ENCODE are those formed between two neighboring genes on the same chromosome [5,6], which are considered by us as RNAs of unannotated genes, but not as chimeric RNAs[1]. Using reverse transcription (RT) and polymerase chain reactions (PCR), we onced tried to verify about three hundreds of reported chimeric RNAs or ESTs that were not associated with known fusion genes, but we could detect none of them, such as the reported CCND1-Trop2 fusion [7,8], in different types of cancer cell lines and tissues, as we mentioned previously [1]. We thus wonder whether the majority of the putative chimeras documented in various databases or in the literature are technical artifacts[1,9] that may mislead the ribonomic research and lead to a great waste in time, resources and effort.

A chimeric RNA can only be derived from one of the two mechanisms, in our opinion[1,3]. One is that the fusion between the two partner genes occurs at the DNA level, i.e. having a fusion gene in a chromosome. So far there have only been about 1,000 fusion genes detected with traditional but reliable techniques such as fluorescent in situ hybridization (FISH)[10], although there are likely more to be found in the future, mainly from cancer cells. However, modern RNA deep sequencing technology has suggested about 10,000 fusion genes without really confirming their true existence at the DNA level [11]. The other mechanism is trans-splicing that joins two RNA molecules together and thus occurs at the RNA level without a DNA basis, i.e. without a corresponding fusion gene in a chromosome. What is debatable is that a large number, probably the majority according to the ENCODE [5,6], of chimeric RNAs are formed between two neighboring genes on the same chromosome. The problem is that no information has been provided on whether these chimeras are formed by trans-splicing of two RNA molecules that were separately transcribed from the two neighboring genes or by cis-splicing of a single RNA molecule that was transcribed via a so-called “read-through mechanism”, i.e. transcription reading from the upstream gene through the downstream one. In our opinion, if the latter (i.e. the “read-through” mechanism) is the case, the cis-spliced RNA should be considered as a product of an unannotated gene, but not as a chimera, because in most aspects these unannotated genes are the same as the canonically annotated ones. Therefore these RNAs should be studied as usual, but not as special cases like chimeras[1,3]. This definition proposed by us culls away this largest portion of reported chimeras from the chimeric RNA category[1,3], thus greatly shrinking the size of chimeric RNA family.

The human mt genome is a circular DNA of about 1.65 kilo-base-pairs (kbs) and is transcribed from both strands of the DNA double helix. The transcript from the inner light strand (L-strand) encodes the ND6protein and eight mt-tRNAs. Transcription from the outer heavy strand (H-strand) could be initiated from two different sites, yielding a short transcript and a long one[12,13]. The short one will be cleaved to the 12S rRNA and the 16S rRNA only, while the long one is almost as long as the whole mtDNA and will be cleaved to the two mt-rRNAs, 12 mt-mRNAs and 14 mt-tRNAs [12–14].

Because mtDNA is transcribed from both strands, it produces both sense and antisense that may anneal to andprime each other not only in PCR but also in consecutive RTs [1]. The human mtDNA not only has many repeated regions but also has a large number of homologous sequences in the nuclear genome [3]. Because each cell has hundreds or even thousands of mitochondria, and each copy of mtDNA may be expressed to multiple copies of RNA, these repeats and homologues of sequences and their sense-antisense pairs are amplified, at the RNA level, at least hundreds or thousands of times in each cell. These features lead us to a hypothesis that chimeric cDNAs might be easily formed by mt sequences as SHS-containing artifacts during RT or PCR. Our overarching hypothesis is that most of those chimeric cDNAs that contain an SHS but are not associated with a fusion gene in a chromosome may be technical spuriousness because the SHS mis-primes RT or PCR[1]. Mt-sequence containing chimeras may be a good tool to test our hypothesis, because mt-sequences may have a higher chance to fuse together artificially during RT or PCR. Indeed, we previously found 57 chimeric, trimeric and tetrameric ESTs that contained one or two mt-sequences; in these 57 fusion ESTs mt-sequences appear 63 times in total (calculated from the table 3 of reference [3]), i.e. about 3.8 times per 1-kb DNA, since mtDNA is about 16.5 kbs in length. If nuclear DNA derived fusion ESTs or RNAs appear at this frequency, the 3.1-billion bps of the human genome should appear 11.78-million times in fustion ESTs or RNAs, or in 5.89-million chimeras since each chimera contains two sequences. The fact that currently the number of reported chimeras is much less than 5.89 millions supports our conjecture that mt-sequence has a higher chance to appear in fusion cDNAs.

## Materials and Methods

### Cell lines and cell culture

Cells used in this study included human embryonic kidney epithelial cell line HEK293, human cervical cancer cell line Hela, human prostate cancer cell line PC-3, human breast cancer cell line SKBR-3, andmouse pancreatic cancer cell line Ela-mycPT1 that was established by us from a pancreatic cancer from an Ela-myc transgenic mouse[15,16]. The cells were cultured as routine in an incubator supplied with 5% CO_2_ and in a DMEM supplemented with 10% fetal bovine serum.When reaching about 80% confluence, the cells were harvested with a gentle trypsin digestion.

### RT, PCR and DNA sequencing

Total RNA was extracted from the cells using Trizol and was subject to DNase I digestion to remove DNA residual, followed by inactivation of the enzyme as described before[9,17,18]. The RNA sample(3 μg) was then primed with random hexamers in a volume of 20-μl RT to generatea cDNA library. For determination of RNA polyadenylation, RT was also primed with our newA primer that consisted of 17 dTs and an artificial sequence, which is the newC primer (Table 1). PCR was performed with the indicated primers (Table 1) and with 1 μl of RT product as the template. To ensure that mtDNA had been removed from the RNA sample and thus RT-PCR amplified only cDNA, but not also mtDNA, the same amount (3 μg) of RNA was diluted to 20 μl, 1 μl of which was directly used, without RT, as the template in a control PCR(non-RT control). PCR products were fractioned in 1% agarose gel and visualized with ethidium bromide (EB) staining. The DNA fragment of interest was excised out and purified from the gel by centrifugation as detailed before[19], followed by being clonedinto a T-A vector[18,20–22]. The resultant plasmids were sequenced by Genewiz Inc. (South Plainfield, NJ). To further confirm the identity of a cDNA, sometimes the purified DNA fragments were also directly sequenced without being cloned.

**Table 1:** Primers used in this study.

Primer Name	Sequence
NewA	5'-GTGGAGTCTACGCGAACTTGTCC**T**_**17**_-3'
NewC	5'-GTGGAGTCTACGCGAACTTGTCC -3'
mtF2006	5'-GGTGATAGCTGGTTGTCCAA-3'
mtF2847	5'-GTACATGCTAAGACTTCACC-3'
mtR2694	5'-AATTGACCTGCCCGTGAAGA-3'
mtF2581	5'-ACCGTGCAAAGGTAGCATAATCACT-3'
mtR3071	5'-GAACTCAGATCACGTAGGAC-3'
mtF3168	5'-CCGTAAATGATATCATCTCAACTT-3'
898652F4	5'-TTGGTTCTCAGGGTTTGTTA-3'
898652R715	5'-TTCACCTCTCCTGGCCATGG-3'
mBCL2-FL1211	5'-CCGGGAGAACAGGGTATGAT-3'
mBcl2-R2i	5'-CTCATTCAACCAGACATGCAC-3'

### cDNA protection assay

To verify the true existence of chimeric RNAs, cDNA protection assay was performed as we detailed before [9]. Strand-specific DNA oligos were in vitro synthesized using PCR with one biotinylated primer, followed by capture with streptavidin-coated magnetic beads [23]. These single-stranded DNA oligos were used not only as probes to protect the RNAs but also as one of the positive controls in the PCR step of the protection assay, because of the lack of true positive control, since all cell lines that were detected by RT-PCR to express a chimeric RNA were the ones to be studied.

### Isolation of mtDNA

We developed a simple method for isolation and purification of mtDNA from cultured cells. Briefly, 100,000 or more cells were collected from a Petri dish by gentle digestion in 1.5 ml of trypsin solution, followed by a quick centrifugation in an Eppendorf tube using a mini-centrifuge. The cell pellet was washed twice, each with 1.5 ml of phosphate buffered saline. After adding 35 μl of H_2_O, 50 μl of 1:1 phenol-chloroform, and 8 μl of the 6x DNA loading dye routinely used for loading DNA into agarose gel, the cell pellet was mixed with a vortex mixer at the highest speed for 60 seconds, followed by centrifugation in a mini-centrifuge for 2 minutes to precipitate cell debris. The aqueous supernatant was transferred into a well in a thick 1% agarose gel containing EB, followed by electrophoresis and photographing the gel. If the well in the agarose gel was not deep enough to accommodate all the aqueous supernatant, the supernatant could be concentrated first using ethanol precipitation; the mtDNA was then suspended again in a smaller volume (about 20 μl) of H_2_O before being loaded into the gel.

### mtDNA references

There are many mtDNA sequences in different databases and these reference sequences differ slightly from one another. The AY195786.2 and the mtDNA used in the UCSC Genome Browser (www.genome.ucsc.edu, UCSC 2014 updated, GRCh38/hg38), which differ slightly between each other, were mainly used in our analyses of the mt sequences unless specified.

## Results

### Cloning of chimeric cDNAs in which two 16S rRNA sequences join at an SHS

**Fig 2:**
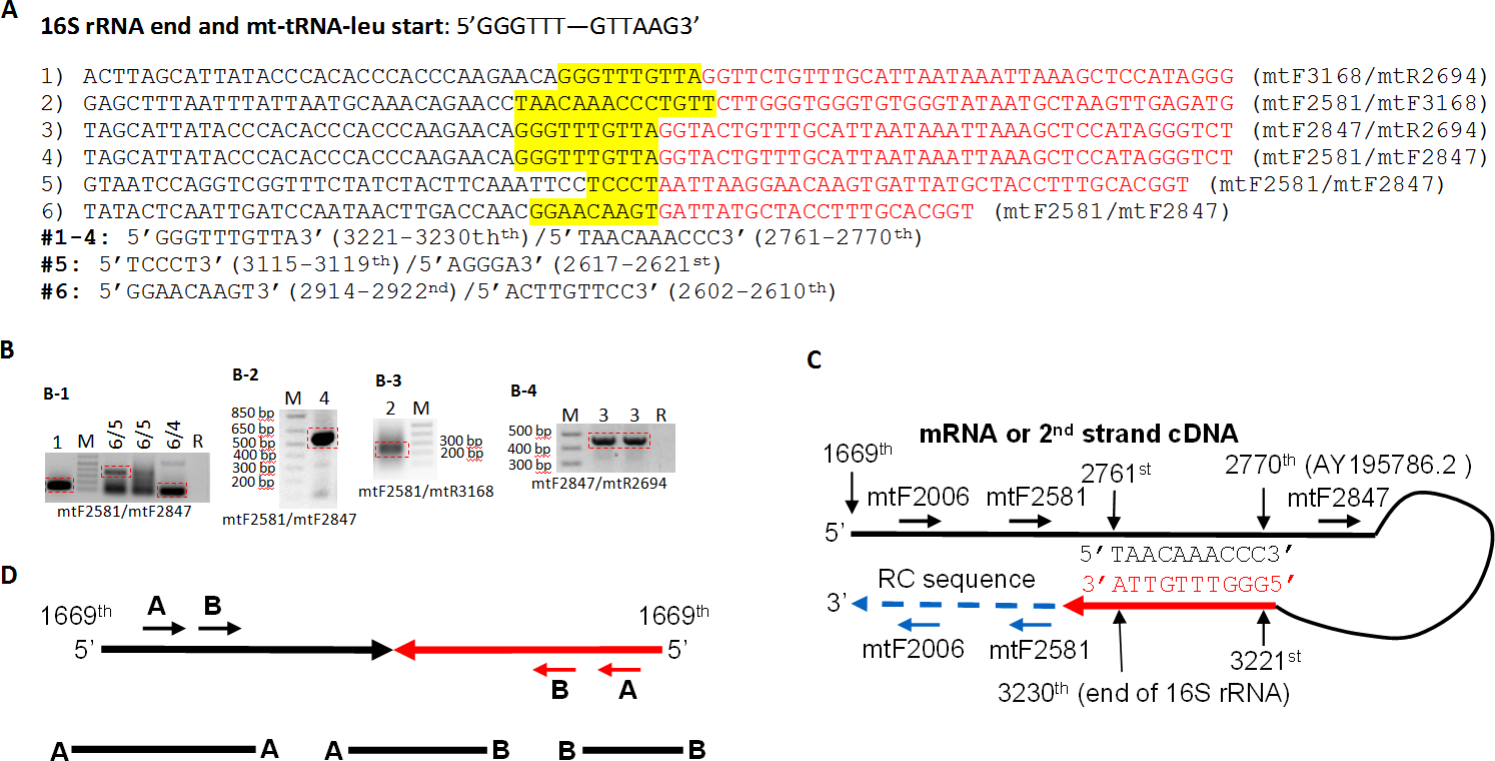
Illustration of the six 16S rRNA chimeric RNAs we cloned. **A**: The human 16S rRNA ends with 5’GGGTTT3’, followed by the mt-tRNA-leu that starts with 5’GTTAAG3’. The region around the joining site of the two partner sequences in the six 16S rRNA chimeras we cloned is presented, with the 5’ and 3’ partner sequences shown in black and red colors, respectively. The primers used for the cloning are in parenthesis. The two partners overlap at an SHS that is shaded by yellow color. In the top four chimeras, the 5’ and 3’ partner sequences overlap at 5’GGGTTTGTTA3’, which is the 2761-2770th/3221-3230^th^ bps of the mtDNA. In the 5^th^ chimera, the two partner sequences overlap at 5’TCCCT3’ (the 3115-3119^th^/2617-2621^st^), whereas in the 6^th^ chimera the two partner sequences overlap at 5’GGAACAAGT3’ (the 2914-2292^nd^/2602-2610^th^). All the nt numbers are referred to AY195786.2. All the overlaps are in a mutually reverse-complementary manner. **B**: PCR was performed with RT product as the template and with the primers indicated. The PCR product was loaded into an agarose gel. M indicatesmarkers of DNA molecular weight, which in B-1 were the 100-bp, 200-bp, 300-bp, 400-bp, 500-bp and 650-bp from the bottom. R in B-1 and B-4 indicates a non-RT control, i.e. a PCR with a diluated RNA sample directly used, without RT, as the template. The numbers 1 to 6 indicate the chimeras shown in **A**. The chimeric DNA band in the red box was excised out from the gel for purification and cloning. **C**: Illustration of our hypothesis of how the first four chimeras may be formed as artifacts, because the SHS mis-primes RT or PCR and uses the 1669-2760^th^ nts of the 16S rRNA (or the second strand cDNA) as the template to synthesize a reversely complementary (RC) sequence, resulting in a palindromic or hairpin-like cDNA. Positions of some primers (mtF2006, mtF2581 and mtF2847) on the cDNA are indicated. **D**: Because of the palindromic nature, a single primer coined as A or B may serve as both forward and reverse primers in PCR to amplify the chimera. Using two primers with the same orientation, say A and B, may produce 3 bands of different sizes primed respectively by the A-A pair, A-B pair, and B-B pair, although sometimes only 2 bands may be discerned easily.

We used different pairs of primer specific to the 16S rRNA to amplify, in PCR, random-hexamer-primed RT products of total RNA samples from Hela, HEK293, PC-3 and SKBR-3 cells. The PCR products were purified from agarose gel and then cloned into a T-A vector. The anticipated DNA fragments were all abundant and easily visiblein agarose gel after 30 cycles of PCR, but we performed 35 cycles to make cloning easier.Of the bacterial colonies from each cloning procedure for each pair of primer and each cell line, 3 to 5 colonies were sequenced, which identified a total of 6 chimeric cDNAs that were formed by two regions of the 16S rRNA (Fig 2A and S1 File). Fig 2B shows typical images of these chimeras visualized in agarose gels, but the density of the bands varied greatly among different experiments and different cell lines, in part because the number of mitochondria per cell and the expression of 16S rRNA differed greatly among different cell lines while we did not intend to study the expression level. All chimeras appeared in all cell lines studied. Analyses of these chimeras reveal that 4 of the 6 have the last 6 nts of the 16S rRNA plus the first 4 nts of the tRNA-leu, i.e. the 3221-3230^th^ (5’GGGTTTGTTA3’) bps of the mtDNA with the AY195786.2 as the reference, reversely complementary to the 2761-2770^th^ (5’TAACAAACCC3’) region of the mtDNA that belongs to the 16S rRNA (Fig 2B). This 10-nt homologue constituted an SHS, which presumably allowed the end of the 16S rRNA to loop back and anneal to the 2761-2770^th^ nts. The 5’GGGTTTGTTA3’ sequence can thus prime RT or PCR with the 5’-part of the 16S rRNA or the second strand of the cDNA as the template (Fig 2C). The product is a palindromic sequence that may form a hairpin-like structure of a 1102-bp (the 1669-2770^th^ bps of the mtDNA) double-stranded stem and a large (450 nts) single-stranded loop (Fig 2C). Because these chimeras contain a palindromic region, a single primer, either a forward one or a reverse one, or two primers with the same orientation, may be used in PCR to amplify the chimeric cDNA (Fig 2D). Actually, some of the sequenced clones wereinitially amplified using two forward primers, which usually produced two to three bands in the gel that were actually the same chimera with different lengths (Fig 2D).

In the 5^th^ chimeric sequence we cloned, the 2617-2621^st^ (5’AGGGA3’) bps of the mtDNA were reversely complementary to the 3115-3119^th^ (5’TCCCT3’) bps of the mtDNA (in AY195786.2). However, the 3’-partner of this chimera has a complete homologue on chromosomes (chrs) 8, 5 and 3, and therefore this may also be a nuclear-mt chimera instead, just like some nuclear-mt chimeric ESTs we previously identified from the NCBI database [3]. The 6^th^ chimera we cloned might be formed by two 16S rRNA sequences, in which the 2602-2610^th^ (5’ACTTGTTCC3’) bps of the mtDNA were reversely complementary to the 2906-2922^nd^ (5’GGAACAAGT3’) bps of the mtDNA (in YA195786.2), constituting an SHS between the two partner sequences. However, the 3’ partner sequence has one complete homologue at chrs 4, 5, 8 and 10 and two complete homologues on chr 3; thus this may also be a nuclear-mt chimera instead.

### Cloning of an ATP8 containing chimeric cDNA

**Fig 3:**
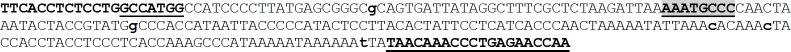
Cloning an ATP8-sequence containing chimera. Our 898652R715 primer (the first 20 nts in boldface) has its last 7 nts (underlined) annealed to and mis-priming the amplification of the 569382-569448^th^-bp region of chr 1 or the 8829-8895^th^-bp region of the mtDNA (AY195786.2) with 1-nt mismatch (the lowercase letter “g” in boldface) as the 5’ partner. The 3’ partner is the 8360-8521^st^-bp region of the mtDNA (AY195786.2), with our forward primer at the 3’ terminus (898652F4; the underlined 20 nts in boldface) in a reverse-complementary manner. The 3’ partner may also be the 633501-633694^th^-bp region of chr 1 with mismatches at 4 nts (lowercase letters in boldface). The two partners overlap at 5’AAATGCCC3’ (underlined, boldfaced, and shaded in grey), which are the 8888-8895^th^-bps/8360-8367^th^-bps of the mtDNA in AY195786.2.

We tended to verify the EST BE898652 that is an mt-sequence containing chimera, but we was not able to detect it from the cell lines we used. Instead, we inadvertently cloned a chimeric cDNA from Hela cells that was amplified in PCR because our reverse primer has its last 7 ntsidentical to the 5’ partner sequence, which makes this primer function as both forward and reverseprimers (Fig 3). The 5’ partner completely matches to the 634002-634068^th^ bps of chr1but may alsobe the 8829-8895^th^ bps of the mtDNA (in AY195786.2) with 1-nt mismatch, which is part of the ATP6 sequence (GeneID: 4508). The 3’ partner iscompletely matched to the 8361-8522^th^-bp region of the mtDNA that contains the last 2 nts of the mt-tRNA-Lys while the remaining belongs to the ATP8. It is more likely that the ATP8 partner is an RNA variant starting from the penultimate nt of mt-tRNA-Lys. Therefore, this cDNA is mostly likely to be a chr 1-ATP8 chimera with two partners overlapped at the 5’AAATGCCC3’/5’AAATGCCC3’ (Fig 3).The second possibility is that this is an ATP8-ATP6 RNA with 1-nt mismatch at the ATP6; in this casethe 5’AAATGCCC3’/5’AAATGCCC3’ overlapping sequences are the 8888-8895^th^-bp/8360-8367^th^-bp regions of the mtDNA in AY195786.2 at the same orientation. Moreover, it also remains possible, although less likely, that the 3’ partner may be the 633501-633694^th^-bp region of chr 1 with four single-nt mismatches (Fig 3).

### Bioinformatic analysis of chimeric ESTs with mt-sequences as both partners

**Fig 4:**
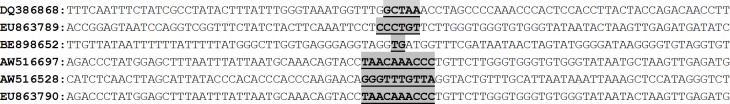
Part of the sequence around the joining site of the six ESTs with mt sequences as both partners. In each EST, the SHS is shaded in grey color. In the BE898652, the two partner sequences are at the same orientation and overlap at only 2 nts. In the AW516697, AW516528 and EU863790, the two partners overlap at 5’GGGTTTGTTA3’ or its reversely complementary sequence.

We used the UCSC Genome Browser to analyze six chimeric ESTs we previously identified from the NCBI database [3] that were formed between two mt-sequences (Fig 4) and had been well studied [24–31]. The U863789.1 [25] has the 2810-3125^th^ bps of mtDNA as its 3’ partner and the 1673-3227^th^ bps of mtDNA in the reverse complementary manner, i.e. in the 3227-1673^rd^ orientation, as its 5’ partner, in the reference mtDNA used by the UCSC that differs slightly from AY195786.2. The two partners have 6 nts (5’CCCTGT3’/3’GGGACA5’) overlapped, which are the 3120-3125^th^-bps/3222-3227^th^-pbs, in a reverse complementary manner. The DQ386868 chimera[27] has the 1718-2537^th^ bps of mtDNA in the reversely-complementary manner, thus in the 2537-1718^th^ orientation, as the 5’ partner and the 1673-3230^th^ bps of mtDNA as the 3’ partner, which in the reference mtDNA of the UCSC genome browseris the annotated end of the 16S rRNA. The two partners overlap at 5 nts (3’CGATT5’/5’GCTAA3’), which are the 1714-1718^th^-bps/1669-1673^rd^-bps. It is likely that in the DQ386868, the 16S rRNA ends at its annotated site without polyadenylation. Interestingly, the DQ386868 forms a long hairpin-like structure that contains an 820-bp region of double-stranded palindromic sequence and a 45-nt-long single-stranded loop, which, as having been illustrated for this [27] and for several other chimeric RNAs [25] by the authors, is similar to what we illustrate in Fig 2C. The sequence around the joining site in AW516697 is actually the same as that in the EU863790 but is reversely complementary to that in the AW516528, obviously because the latter was sequenced from the other end, all with the two partners overlapping at 5’GGGTTTGTTA3’ or its reversely complementary sequence. Therefore, the last three ESTs are considered the same as the first four 16S rRNA chimeras we cloned.

In five of these six 16S rRNA containing chimeric ESTs, the 5’ and 3’ sequences join at an SHS, just like the six chimeras we cloned. However, in the remaining one, i.e. the BE89652, the two partners overlap only at 2 nts and are at the same orientation of the mtDNA, i.e. from the same strand of the DNA double helix. Although we were not able to verify the existence of the BE89652 by RT-PCR in the cell lines we studied, the ATP8-containing chimera we cloned is similar to it.

### Identification of 16S rRNA ends with or without a poly-A tail

**Fig 5:**
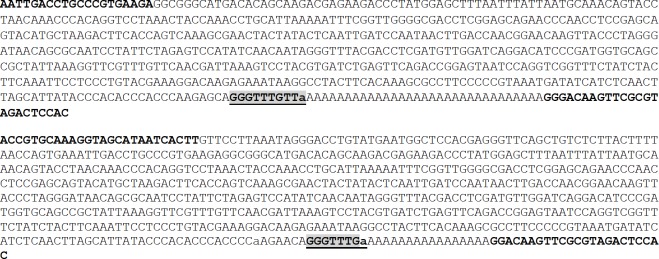
Two different ending sites (shaded, boldfaced and underlined) of 16S rRNA with the lowercase ‘a” belonging to either the RNA or the poly-A tail appended during polyadenylation. The boldfaced sequence at the 5’ terminus is the mtR2694 (top panel) or the mtF2581 (bottom panel) primer. The boldfaced sequence after the poly-A tail is the newC primer or part of the newA primer.

In four of the above-described six 16S rRNA containing chimeras we cloned, the two partner sequencesjoinat the 5’GGGTTTGTTA3’, with the last “A” as the 4^th^ nt of the tRNA-leu[32]. Since none of the plasmid clones we sequenced had the joining site at the annotated end of 16S rRNA, we suspected that the annotated end of 16S rRNA is not a common one, which is partly supported by the reports that 16S rRNA has alternative ends around 5’GGGTTT3’ and can be extended into the mt-tRNA-leu [32,33]. Since 16S rRNA has both polyadenylated and non-polyadenylated forms[13], we primed RT withour newA primer that consisted of 17 dTs at the 3’ end and a NewC sequence at the 5’ endas a tag (Table 1). The RT product was used as the templatein PCR with a NewA or newC primer paired with a 16S rRNA specific primer to amplify the polyadenylated form of 16S rRNA, followed by cloning it into a T-A vector. One of the five plasmid clones we sequenced showed that the 16S rRNA ended with 5’GGGTTTG3’, with the last “G” being the first mt-tRNA-leu nt (the 3227^th^ nt of mtDNA in theAY195786.2), while the remaining four clones ended with 5’GGGTTTGTTA3’ (Fig 5). Although it remains possible that the last “A” might beappended by polyadenylation, the above-described chimeric sequences suggest that it is more likely to belong to the RNA. These sequences, together with the above-described four chimeras we cloned and the last three ESTs shown in Fig 4, lead us to a conclusion that 16S rRNA usually ends at the 4^th^ nt of tRNA-leu. However, ending at the annotated site, i.e. the last T of 5’GGGTTT3’, or at the first nt of tRNA-leu (i.e. the last “G” of the 5’GGGTTTG3’), as shown in Fig 5, did occur but asa much less common event, likely to provide the cells with a mechanism to diversify the functions of 16S rRNA in different situations.

### Earlier-ending16S rRNA with mismatches at As and Cs

**Fig 6:**
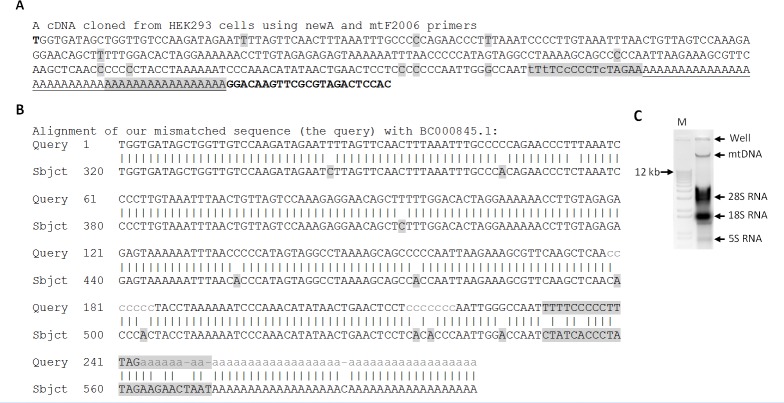
Cloning an earlier ending of 16S rRNA with 11 mismatches and a poly-A tail. **A**: Using our newA and mtF2006 primers in PCR to amplify the newA-primed RT product from HEK293 cells, we obtained a cDNA that contains the 2006-2274^th^-bp region of the mtDNA in AY195786.2, followed by a 16-nt sequence (shaded) that has four mismatches (in lowercase letters) to all mtDNA references we can find, and then a poly-A tail (underlined). Our newA primer contains 17 As (shaded), which constitute part of the poly-A tail, and a 23-nt sequence that is our newC primer (boldfaced). The 2006-2274^th^-bp region contains 11 single-nt mismatches (shaded). **B**: Alignment of our sequence with BC000845.1, which is a normal mt sequence and is 24-nt longer (shaded) than our sequence before a poly-A tail. Note that within the 2006-2274^th^-bp region, the 11 mismatches (shaded) occur only at A and C, compared with the BC000845.1 or with all mtDNA references we can find. **C**: Image of mtDNA isolated from HEK293 cells using the simple method we developed. The mtDNA that migrated much slower than the 12-kb band in the linear DNA markers (M) in a 1% agarose gel was excised from the gel and purified for PCR, cloning and sequencing.

During cloning poly-A tail of 16S rRNA with our mtF2006 and newA primers, we obtained one plasmid clone from HEK293 cells that might end at the 2274^th^ bp of the mtDNA (AY195786.2) followed by a 16-nt sequence that is highly (4/16) mismatched to any mtDNA reference we can find (Fig 6A). This 16-nt sequence might either be a highly mutated region orbe appended by an RNA polymerase in the cell via a non-template mechanism, because it was followed by a poly-A tail appended by polyadenylation that should occur inside the cell. Regardless the nature of this 16-nt region, this clone shows that the 16S rRNA ended earlier and was polyadenylated. Raking various sequence databases, we found an EST, BC000845.1 that ends at a nearby position, i.e. at 2290^th^ bp of the mtDNA, with a poly-A tail (Fig 6B).

Interestingly, in the 2006-2274^th^-bp region, there are 11 single-nt mismatches, all occurring to A (7 A-to-C and 1 A-to-G) and C [3 C-to-U (T)], but none to G or U (T). Sequencing other plasmids cloned from RT-PCR with this and other pairs of primer revealed five additional plasmid clones that contained all 11 mismatches while some other clones did not have any of these mismatches. Peculiarly, we did not obtain any clone that had only some of the 11 mismatches, and we have no explanation for this observation. To determine whether the 11 mismatches occur at the mtDNA or via RNA editing without involving mtDNA, we purified and sequenced the mtDNA from HEK293 cells (Fig 6C), but after sequencing 10 mtDNA samples we did not find any mismatches, confirming that at least some copies of mtDNA in HEK293 cells are normal. Since each cell has hundreds to thousands of mitochondria, it is possible that other mitochondria do not have these mutations or do not perform RNA editing at these sites.

Also, it is interesting that all three C-to-T mismatches occurred after a T and resulted in a sequence of consecutive Ts. Similarly, 7 of the 8 mismatches at A occur after or before a C, resulting in a sequence of consecutive Cs, whereas one A-to-G mismatch converts the sequence to GGGCCC. The meaning of these consecutive-T and -C sequences is unclear.

We also found that the 3194^th^ bp of the mtDNA in AY195786.2, which is the 3198^th^ bp in the mtDNA reference of the UCSC genome browser (5’aacttag**T**attatac3’), is a T-to-C (T3194C) mismatch, either a polymorphism or a mutation, in at least SKBR-3 and PC-3 cell lines.

### Cloning of a mouse Bcl2-Nek9 chimeric cDNA

**Fig 7:**
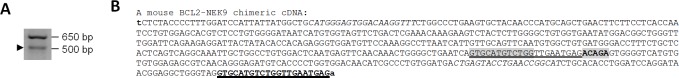
Cloning of a mouse Bcl2-Nek9 chimeric cDNA. **A**: Using Bcl2-L1211 and Bcl2-R2i primers in PCR to amplify an RT product from Ela-mycPT1 cells resulted in not only the anticipated band of the Bcl2-β mRNA variant at the top but also a smaller band of about 500 bps (arrowhead). **B**: Purification, cloning and sequencing of the smaller band (indicated by the arrow in A) reveal that it is a Bcl2-Nek9 chimera (with the lowercase “t” and “a” at both ends appended by Taq DNA polymerase). The Bcl2-R2i primer (underlined region inside the sequence) has 12 nts (shaded) matched to the Nek9 sequence. There is a 5-nt unmatchable sequence (shaded 5’ACAGA3’ in boldface) after this BCl2-R2i primer. This chimera ends with another Bcl2-R2i sequence (underlined 20 nts in boldface at the 3’ end), but the Bcl2-L1211 forward primer is not in the cDNA.

During our study of the expression of a Bcl2 mRNA variant in our Ela-mycPT1 mouse pancreatic cancer cell line, we serendipitously obtained a chimeric cDNA that was a fusion between the Bcl2 and the Nek9 genes (Fig 7). This cDNA repeatedly appeared in our RT-PCR products visualized in agarose gel[34]. PCR was not able to detect it in genomic DNA, indicating that it was not associated with a fusion gene. Sequence analysis suggests that our reverse primer Bcl2-R2i has 12 nts at its 3’ terminus reversely-complementary to a region of the Nek9 mRNA, and thus primes the Nek9 amplification. The Bcl2-R2i sequence appears at both ends of the Bcl2 partner. There is a 5-nt unmatchable sequence appended to the 3’ end of the inner Bcl2-R2i sequence, which together with part ofthe inner Bcl2-R2i constitutes a gap sequence between the two partners. However, the forward Bcl2 primer was not in the cDNA, making it unknown how this cDNA is formed and amplified.

### PCR primed by annealing at only four nts

**Fig 8:**
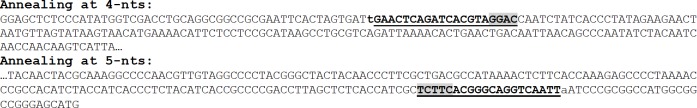
Part of the cDNAs cloned via a primer annealing only at 4 or 5 nts during PCR. **Top panel**: a cDNA was inadvertently cloned because the last 4 nts, i.e. 5’GGAC3’ (shaded), of our mtR3071 primer (underlined and boldfaced) are reversely complementary and thus annealed to the 2263-2266^th^ bps of the mtDNA (AY195786.2). The lowercase letter “t” at the 5’ side of the mtR3071 sequence was appended by Taq DNA polymerase, and the sequence before this “t” belongs to the T-A vector. **Bottom panel**: A cDNA was accidently cloned because the last 5 nts, i.e. 5’TCTTC3’ (shaded) in the mtR2694 primer (underlined and boldfaced), is reversely complementary and thus mistakenly annealed to the 3550-3554^th^-bp region of the mtDNA (AY195786.2). The lowercase letter “a” after the primer was appended by Taq DNA polymerase, whereas the sequence after the “a” belongs to the T-A vector. (“…” sequence omitted)

RT-PCRamplification of an RNA sample from HEK293 cells with our NewA and mtR3071 primers followed by T-A cloning resulted in a plasmid clone containing the 2266-3071^st^-bp region of mtDNA (AY195786.2). Sequence analysis suggests that the cDNA is amplified because the last 4 nts (5’GGAC3’) of our mtR3071 primer are identical, and thus mistakenly annealed, to the 2266-2269^th^ bps of the mtDNA (Fig 8). To our knowledge, this is the first experimental evidence showing that annealing at only 4 nts can prime DNA polymerization. Moreover, in another plasmid clone amplified from SKBR-3 cells with our mtF2847 and mtR2694 primers, the cDNA is amplified because the last 5 nts of our mtF2694 primer are identical and thus mistakenly annealed to the 3550-3554^th^ region of the mtDNA that belongs to the tRNA-leu (Fig 8). Cloning this 2847-3554^th^ region of mtDNA also indicates that there might exist some uncleaved 16S RNA-tRNA-leu transcript.

## Discussion

### Can 16S rRNA and mt-tRNA-leu be produced from the same mt transcript?

In three of the five 16S rRNA containing chimeric ESTs we culled from the NCBI database, one of the two partner sequences ends at the 4^th^ nt of the mt-tRNA-leu, whereas in only one of the five one partner sequence ends as annotated (Fig 4). In four of the six chimeras we cloned (Fig 2), the 16S rRNA ends at the 4^th^ nt of the mt-tRNA-leu as well. Cleavages at the annotated site and other sites, including the one deep into the mt-tRNA-leushown ina sequence in Fig 8, do occur but as much less common events. Therefore we conclude that 16S rRNA needs to be reannotated and the transcript more often ending at the 4^th^ nt of the mt-tRNA-leu should be considered as the wild type. It remains possible that this dominant form of 16S rRNA with four additional nts is produced mainly from the L-strand transcript, but currently we have no inkling about this possibility. If it is produced from an H-strand transcript, a question is raised as to whether the remaining transcript can still be used to produce an mt-tRNA-leu that is 4 nts shorter. There are two possibilities under this scenario: one is that mt-tRNA-leu is often made to be 4nt-shorter as well. If this is the case, it triggers more questions as to whether the shorter mt-tRNA-leu variant functions normally just like the annotated one or it functions differently. The other scenario is that the H-strand transcript, after the longer 16S rRNA is nipped off, is no longer used to produce the mt-tRNA-leu, although it may still be used to produce other mt-RNAs. In other words, the annotated mt-tRNA-leu and the dominant form of 16S rRNA are produced from different H-strand transcripts, which is very wasteful. It is also possible that the mechanism for producing these two neighboring RNAs resembles that for producing ATP8 (the 8366-8572^th^-bps of the mtDNA in gene ID 4509) and ATP6 (the 8527-9207^th^-bps in gene ID 4508) that overlap at 46 nts, i.e. at the 8527-8572^th^-bps. All these possibilities, scenarios or questions deserve further studies.

Our results also confirm that 16S rRNA has both polyadenylated and non-polyadenylated forms. However, although the 16S rRNA region has been reported to have tens of poly-A sites [35,36], we only observe several ending sites around the annotated end with or without polyadenylation, besides an additional site deep into the mt-tRNA-leu. From Fig 2C, one can infer that a spurious chimeric cDNA may form only when a 16S rRNA ends within an SHS, especially at the 5’GGGTTTGTTA3’ region, and without being polyadenylated. In other words, because 16S rRNA has multiple ending sites andmay be polyadenylated, we may not see too many spurious chimeras formed by 16S rRNA.

### Are some cDNAs we cloned mt-mt or mt-nuclear chimeras?

There are four possibilities for the origin of the ATP8-containing cDNA we cloned (Fig 3). First, it is most likely to be a chr 1-ATP8 chimera with the two partners reversely complimentary at the 8-nt SHS, since the two partner sequences match 100% to the corresponding references. What is still unknown is whether this chimera is an authentic RNA or an artificial cDNA derived from SHS-caused mis-priming in RT or PCR. Actually, similar to this chimera, some of the 16S rRNA chimeras we cloned may be nuclear-mt chimeras as well, because one mt-partner has one or more homologues in the nuclear genome. As we have discussed before[3], even if just one of these RNAs is authentic, serious concernsare raised in ribonomics as to how nuclear and mt RNAs can join together and in where of the cell the fusion occurs.Second, it may be an authentic ATP6-ATP8 RNA, as its 5’ partner only has 1-nt mismatch to the ATP6, and this mismatch may be a polymorphism or a mutation. This ATP6-ATP8 RNA may be a cis-spliced product from a L-strand transcriptwith 308 nts (the 8521-8828^th^ bps of the mtDNA) nipped off, but not from an H-strand transcript becauseon the H-strand the ATP8 locates at the upstream of the ATP6. However, this conjecture opposes the well accepted notion that the L-strand encodes only ND6 protein and eight mt-tRNAs[12–14]. We have previously identified seven ESTs that are suspected to be cis-spliced products of mt transcripts and have cloned a 16S rRNA variant that is likely produced from a cis-splicing mechanism [3]. These lines of information and this ATP6-ATP8 cDNA together question the well-accepted notion that mt-transcripts do not undergo cis-splicing [12–14]. Third, even if it is an ATP6-ATP8 RNA, it may also be a chimera, but not a cis-spliced product, that is formed by two mt sequences with the same orientation via a hitherto unknown mechanism. Alternatively, like the chr 1-ATP8, this chimera may also bea technical artifact created by mis-priming at the 8-nt SHS. However, because the two partners have the same orientation, the artifactis formed during PCR, but not RT. This is because only PCR can generate the second strand of cDNA that anneals to the first-strand at the 8-nt SHS and primes the elongation of DNA polymerization to both directions to form a chimera, as we illustrated and explained in detail previously [1,9]. No matter it is an authentic or a spurious chimera, its ATP8 partner may start from the penultimate nt of the mt-tRNA-lys, somewhat similar to the longer 16S rRNA form that extends into the mt-tRNA-leu.

### The chimeric cDNAs we cloned might be technical artifacts

Our overarching hypothesis is that most of those chimeric RNAs that have an SHS but are not associated with a fusion gene in a chromosome are technical artifacts formed by SHS-caused mis-priming in RT or PCR. Obviously, this hypothesis cautionsagainst the use of any techniquethat involves a DNA polymerase, including RNA sequencing, as the main approach to identifying chimeric RNAs. For this reason we have developed a cDNA protection assay in which a cDNA is protected by an RNA and then is PCR-amplified to greatly increase the sensitivity[9]. We performed cDNA protection assay to verify the true existence of all the chimeric RNAs presented in this paper, but were not able to detect any of them. In our opinion, all techniques other than our cDNA protection assay are not reliable for verification of the true existence of a chimeric RNA. However, if the chimera does not really exist, our cDNA protection assay will lack a good positive control and thus cannot verify a true negative result. The modern RNA sequencing technology has hitherto identified a large number, about 10,000, of putative chimeras [11], simply because this technique has the least reliability in this regard, in our opinion. In situ hybridization used in some reported studies [25–27] does not provide sequence information to confirm that both partner sequences form a single target of the probe, but not each alone hybridizes to the probe. We tend to consider that all the cDNAs we presented in this paper, including the six 16S rRNA containing chimeras(Fig 2), are technically spurious. However, we acknowledge that our cDNA protection assay is still less sensitive than routine RT-PCR approach, and that lack of evidence is not evidence of lack. Thereforeour conclusion still awaits concrete evidence. Hopefully, a technique will soon be available that is not only much more sensitive than our cDNA protection assay but also highly reliable.

### Four-nt priming suggests many more artificial cDNAs than we have realized

Even if our overarching hypothesis is correct, one associated question is how short an SHS can stilleffectively prime DNA polymerization, since an SHS can be as short as only a single nt[3]. A 5-nt SHS should be long enough, since pentamers, i.e. 5-nt oligos, are sometimes used to prime RT or PCR, and we have also amplified an mt-sequence due to mis-priming at 5 nts (Fig 8). In this study, we serendipitously found that if a primer has as few as 4 nts at its 3’ terminus identical and thus mistakenly annealed to a template DNA, it can initiate DNA polymerization, thus for the first time showing that a 4-nt SHS may be sufficient to prime. However, a caveat needs to be given that this result does not necessarily mean that tetramers, i.e. 4-nt oligos, can serve as good RT or PCR primers, because it cannot be ruled out that the remaining 16-nt sequence at the 5’ terminus of our mtR3071 primer also helps the DNA polymerase inbinding to the template andinitiating DNA synthesis. In other words, 4-nt SHSs and tetramers may not be the same thing. Another caveat is that not all homologous sequences will anneal and prime DNA polymerization, and therefore intentionally designing an experiment using a 4-nt SHS to prime may or may not work. Nevertheless, this finding is meaningful, as it leads us to a reasonable assumption that the number of artificial cDNAs, chimeric or not, may be many more than we have realized, because homologous regions as short as 4 nts in the nuclear and mt genomes should be many more in number, relative to 5-nt or longer sequences.

### The Bcl2-Nek9 fusion reminds us of unknown mechanisms for priming DNA polymerization

According to our statistics, about 30% of the putative chimeric RNAs and about 26% of the chimeric ESTs in the NCBI database contain an unmatchable sequence as a gap between the two partner genes, which is another category of chimeric RNAs[3]. The Bcl2-Nek9 cDNA we cloned just adds one more tothis largecategory.Why and how a gap is produced in so many RNAs or cDNAsis an intriguing mystery inribonomics. We only identify one (the Bcl2-R2i) of the two primers in one end of the Bcl2-Nek9 cDNA, while neither primer locates at the other end. The Bcl2-R2i primer appears twice in the cDNA, but both copies have the same orientation and part of this primer constitutes part of the gap sequence. These unique features bewilder us as to how this cDNA is formed and amplified frequently in PCR[34]. One critical question is whether a gap sequence is added in by a DNA polymerase via a template-independent mechanism, since, as we have reviewed before [1], many DNA polymerases [37–40,41], such as the human DNA polymerase mu [42], can append one or more nts to the 3’ terminus of a DNA sequence in a non-template manner. Some enzymes can even catalyze 3’-to-5’ polymerization [43,44]. Many researchers use such powerful techniquesas RT, PCR and RNA sequencing without havinga full awareness or giving a detailed discussion of their weaknesses and pitfalls, which greatly worries us. For example, to our knowledge, few researchers who routinely use these techniques know that the technical artifact of so-called exon shuffling, non-canonical trans-splicing or template switching in PCR[45,46] differs from thetemperate-switching by reverse transcriptase in RT[47,48].These shortcomings should be fully considered when these techniques are employed and when a new sequence is obtained. In our opinion, there are many artificial cDNAs in different databases and published studies that have not been fully addressed.

## Conclusions

In this study we cloned eight chimeric cDNAs, of which six contain 16S rRNA, one contains ATP8, and one is a fusion between the Bcl2 and the Nek9, butall these chimeras cannot be detected using cDNA protection assay and thus are likely to be technical artifacts, although currently we still lack technique to futher verify it. Sequence analyses of the six 16S rRNA containing chimerasand several similar chimeric ESTs reveal that they are formed probably because some regions of 16S rRNA are reversely complementary toanother region to form SHSs. The SHS allows the downstream sequence of 16S rRNA to loop back and prime the synthesis of its complementary sequence, resulting in a palindromic sequence that may form a hairpin-like structure. The 16S rRNA gene needs to be reannotated as its dorminant, thus the wild type, RNA is the one ending at the 4^th^ nt of the mt-tRNA-leu,which raises a question as to whether the same H-strand transcript, after it has producedthe longer 16S rRNA, can still produce anmt-tRNA-leu and, if it can, whether the shorter tRNA still functions normally. Moreover, a cDNA was amplified because one primer has 4 nts matched to the template, which inspires us that there may be many more artificial cDNAs than we have realized, because the nuclear and mt genomes should have many more 4-nt than 5-nt or longer homologous regions. The Bcl2-Nek9 chimeric cDNA contains a gap sequence and two copies of a primer in the same orientation but does not contain the other primer. Altogether, the eight chimeric cDNAs we cloned are good examples suggesting that there are many unexpected events that result in artificial cDNAs, in some cases occurring via unknown mechanisms, during RT, PCR or RNA sequencing that involves DNA polymerization. The mtDNA has many repeated regions and many homologues in the nuclear genome, whereas each cell has at least hundreds of copies of mtDNA and each copy may produce many copies of RNA. These features of mt genome and transcriptome make it easy to form SHSs and in turn mt-sequence containing chimeras. A particular cDNA may beanmt-mt chimera in some occasions but an mt-nuclear chimera in other occasions, depending on whether it is the mt or the nuclear RNA or cDNA that stochastically and randomly anneals to a partner at the SHS. Because of this stochastic and random nature of mispriming at an SHS, many chimeric cDNAs are not reproducible or only reproducible sometimes, as we have experienced.

## Supporting Information

S1 File Our mt-chimeric RNA sequences. Sequences of the six 16S rRNA containing chimeric cDNAs cloned from Hela cells using the indicated pair of primers (underlined) that locate at the beginning and the end of the sequence. The yellow-shaded region is the SHS.
(PDF)
