# Supplementary material for: Two RNAs or DNAs May Artificially Fuse Together at a Short Homologous Sequence (SHS) during Reverse Transcription or Polymerase Chain Reactions, and Thus Reporting an SHS-Containing Chimeric RNA Requires Extra Caution
Source: PLoS One. 2016 May 5;11(5):e0154855. doi: 10.1371/journal.pone.0154855 (PMC4858267; doi:10.1371/journal.pone.0154855)
Supplement: S1 File — Sequences of the six 16S rRNA containing chimeric cDNAs cloned from Hela cells using the indicated pair of primers (underlined) that locate at the beginning and the end of the sequence. The yellow-shaded region is the SHS. (PDF) [file pone.0154855.s001.pdf]

Our mt-chimeric RNA sequences:

1) Primers: mtF3168/mtR2694, from Hella cells
CCGTAAATGATATCATCTCAACTTAGCATTATACCCACACCCACCCAAGAACAGGGTTTGTTAGGTTCTGTTTGCAT
TAATAAATTAAAGCTCCATAGGGTCTTCTCGTCTTGCTGAGTCATGCCCGCCTCTTCACGGGCAGGTCAATT

2) Primers: mtF3168/mtF2581, from Hella cells
ACCGTGCAAAGGTAGCATAATCACTTGTTCCTTAAATAGGGACCTGTATGAATGGCTCCACGAGGGTTCAGCTGTCT
CTTACTTTTAACCAGTGAAATTGACCTGCCCGTGAAGAGGCGGGCATGACACAGCAAGACGAGAAGACCCTATGGAG
CTTTAATTTATTAATGCAAACAGAACCTAACAAACCCTGTTCTTGGGTGGGTGTGGGTATAATGCTAAGTTGAGATG
ATATCATTTACGG

3) Primers: mtF2847/mtR2694, from Hella cells
GTACATGCTAAGACTTCACCAGTCAAAGCGAACTACTATACTCAATTGATCCAATAACTTGACCAACGGAACAAGTT
ACCCTAGGGATAACAGCGCAATCCTATTCTAGAGTCCATATCAACAATAGGGTTTACGACCTCGATGTTGGATCAGG
ACATCCCGATGGTGCAGCCGCTATTAAAGGTTCGTTTGTTCAACGATTAAAGTCCTACGTGATCTGAGTTCAGACCG
GAGTAATCCAGGTCGGTTTCTATCTACTTCAAATTCCTCCCTGTACGAAAGGACAAGAGAAATAAGGCCTACTTCAC
AAAGCGCCTTCCCCCGTAAATGATATCATCTCAACTTAGCATTATACCCACACCCACCCAAGAACAGGGTTTGTTAG
GTACTGTTTGCATTAATAAATTAAAGCTCCATAGGGTCTTCTCGTCTTGCTGTGTCATGCCCGCCTCTTCACGGGCA
GGTCAATT

4) Primers: mtF2581/mtF2847, from Hela cells
GTACATGCTAAGACTTCACCAGTCAAAGCGAACTACTATACCCAATTGATCCAATAACTTGACCAACGGAACAAGTT
ACCCTAGGGATAACAGCGCAATCCTATTCTAGAGTCCATATCAACAATAGGGTTTACGACCTCGATGTTGGATCAGG
ACATCCCGATGGTGCAGCCGCTATTAAAGGTTCGTTTGTTCAACGATTAAAGTCCTACGTGATCTGAGTTCAGACCG
GAGTAATCCAGGTCGGTTTCTATCTACTTCAAATTCCTCCCTGTACGAAAGGACAAGAGAAATAAGGCCTACTTCAC
AAAGCGCCTTCCCCCGTAAATGATATCATCTCAACTTAGCATTATACCCACACCCACCCAAGAACAGGGTTTGTTAG
GTACTGTTTGCATTAATAAATTAAAGCTCCATAGGGTCTTCTCGTCTTGCTGTGTCATGCCCGCCTCTTCACGGGCA
GGTCAATTTCACTGGTTAAAAGTAAGAGACAGCTGAACCCTCGTGGAGCCATTCATACAGGTCCCTATTTAAGGAAC
AAGTGATTATGCTACCTTTGCACGGT

5) Primers: mtF2581/mtF2847 from Hela cells
GTACATGCTAAGACTTCACCGGTCAAAGCGAACTACTATACTCAATTGATCCAATAACTTGACCAACGGAACAAGTT
ACCCTAGGGATAACAGCGCAATCCTATTCTAGAGTCCATATCAACAATAGGGTTTACGACCTCGATGTTGGATCAGG
ACATCCCGATGGTGCAGCCGCTATTAAAGGTTCGTTTGTTCAACGATTAAAGTCCTACGTGATCTGAGTTCAGACCG
GAGTAATCCAGGTCGGTTTCTATCTACTTCAAATTCCTCCCTAATTAAGGAACAAGTGATTATGCTACCTTTGCACG
GT

6) Primers: mtF2581/mtF2847 from Hela cells
GTACATGCTAAGACTTCACCAGTCAAAGCGAACTACTATACTCAATTGATCCAATAACTTGACCAACGGAACAAGTG
ATTATGCTACCTTTGCACGGT

Legend: Sequences of the six 16S rRNA containing chimeric cDNAs cloned from Hela cells using the indicated pair of primers (underlined) that locate at the beginning and the end of the sequence. The yellow-shaded region is the SHS.
